# Artificial Pond Habitats Placed in an Australian Berry Farm Support Invertebrate Diversity Including Pollinating Flies

**DOI:** 10.1002/ece3.73423

**Published:** 2026-04-07

**Authors:** Jelena Preradovic, Romina Rader, Blake M. Dawson, Lena A. Schmidt, Raylea Rowbottom, Abby E. Davis

**Affiliations:** ^1^ School of Environmental and Rural Science University of New England Armidale New South Wales Australia; ^2^ Department of Entomology Michigan State University East Lansing Michigan USA; ^3^ SeedPurity Pty Ltd. Margate Australia

**Keywords:** artificial ponds, hoverflies, invertebrate biodiversity, microhabitats, non‐floral resources, pollinating flies

## Abstract

Artificial ponds in agricultural landscapes may provide critical microhabitats for aquatic and semi‐aquatic invertebrates, including pollinating flies that require water for larval development. To test this idea, we deployed eight artificial ponds, comparing water‐only controls with ponds containing decomposed blackberry plant material, at four farm blocks within a commercial berry farm located in New South Wales, Australia. Ponds were inspected every 3–4 days to monitor pond taxa appearance alongside eristaline (Diptera: Syrphidae) fly oviposition, larval development and pupation. Over 43 days, we recorded 40 invertebrate taxa from nine orders; seven taxa were observed only 1–3 times during the 43‐day trial and were not present in the final sampling period. In total, 33 taxa colonised the artificial ponds for the entire trial period including three eristaline flies ( *Eristalis tenax* , *Eristalinus punctulatus* and *Austalis copiosa*). Eristaline fly eggs appeared within 9 days of deployment, larvae within 17 days, and pupation occurred within 38 days of deployment within the adjacent soil and grass. Overall, this study demonstrates that artificial ponds could act as multifunctional habitats that support invertebrate communities and provide a simple, scalable way to enhance on‐farm insect biodiversity while supporting pollinating flies. Future research directions could include broader‐scale designs that maintain eristaline populations while reducing pest taxa.

## Graphical abstract

This study investigated whether small, artificial ponds deployed within a commercial farm can function as microhabitats supporting diverse invertebrate communities. We recorded 40 invertebrate taxa from nine orders utilising the ponds, including honey bees, lady beetles and spiders, as well as three species of eristaline hoverflies. The situation has prompted growers to explore alternative pollinators capable of supporting crop pollination services.

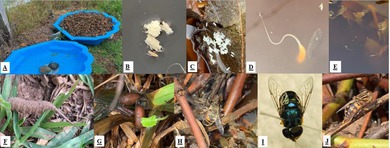

## 1 Introduction

Agricultural intensification has led to the loss of small natural water bodies such as ponds, dams, and river pools, reducing freshwater and semi‐aquatic biodiversity in farmland ecosystems (Biggs et al. 2017; Hill et al. 2025). Pond‐associated insect taxa play a pivotal role in sustaining productive and resilient agricultural ecosystem services by providing pollination, pest suppression, nutrient recycling, and food web stability (Davis, Bickel, Saunders, and Rader 2023; Davis, Schmidt, Harrington, et al. 2023; Davis, Schmidt, Santos, et al. 2023; Dawson et al. 2025; de Paula Malheiros et al. 2025; Kheam et al. 2025). Many insect taxa also utilise aquatic environments to complete their immature stages of development (Hazell et al. 2001; Davies et al. 2016; Simaika et al. 2016; Hill et al. 2025). Small ponds and artificial water bodies can thus act as isolated patches of high‐quality habitat, supporting the ecosystem services beneficial to agricultural land holders (Biggs et al. 2017; Littlefair et al. 2024; Hill et al. 2025).

Among the taxa that benefit from small, shallow water bodies such as ponds and drainage ditches are pollinating flies from the subfamily Eristalinae (Diptera: Syrphidae). Eristaline fly larvae develop in semi‐aquatic environments rich in decaying organic materials (Larson et al. 2001; Nicholas et al. 2018; Campoy et al. 2020; Cao et al. 2022; Davis, Bickel, Saunders, and Rader 2023). Many eristaline flies are also effective crop pollinators due to their requirements for nectar and pollen for reproduction. For example, the European drone fly, *Eristalis tenax* (Linnaeus, 1758), and the golden (Australian) native drone fly, *Eristalinus punctulatus* (Macquart, 1847), are effective crop pollinators of carrot, sweet pepper, pak choy, raspberry, and blackberry crops (Gaffney et al. 2018; Davis, Schmidt, Harrington, et al. 2023). Hence, the creation or restoration of artificial ponds within farms not only promotes greater habitat heterogeneity but could also potentially support broader biodiversity and ecosystem service initiatives (Brainwood and Burgin 2009; Hill et al. 2025). Research has primarily examined biodiversity in permanent farm dams (Dudgeon et al. 2006; Dudgeon 2010; Reid et al. 2019; Fluet‐Chouinard et al. 2023); however, recent studies show that small artificial ponds with decaying plant matter can attract fly pollinators and support their larvae under field conditions (Davis, Schmidt, Harrington, et al. 2023; Dawson et al. 2025).

Here, we deployed four artificial ponds filled with water and decaying plant materials (hereafter called plant‐substrate ponds) and four water‐only controls across four blocks on a commercial berry farm located in the Mid North Coast of New South Wales, Australia, to assess pond colonisation dynamics. We recorded the appearance and diversity of aquatic and semi‐aquatic invertebrates, alongside oviposition, larval and pupation development by pollinating eristaline flies. We ask the following questions:

1. How quickly (in days) do invertebrate taxa colonise artificial ponds in berry cropping systems?
2. To what extent do eristaline flies use the habitat for oviposition, larval development, and pupation?

## 2 Material and Methods

**FIGURE 1:**
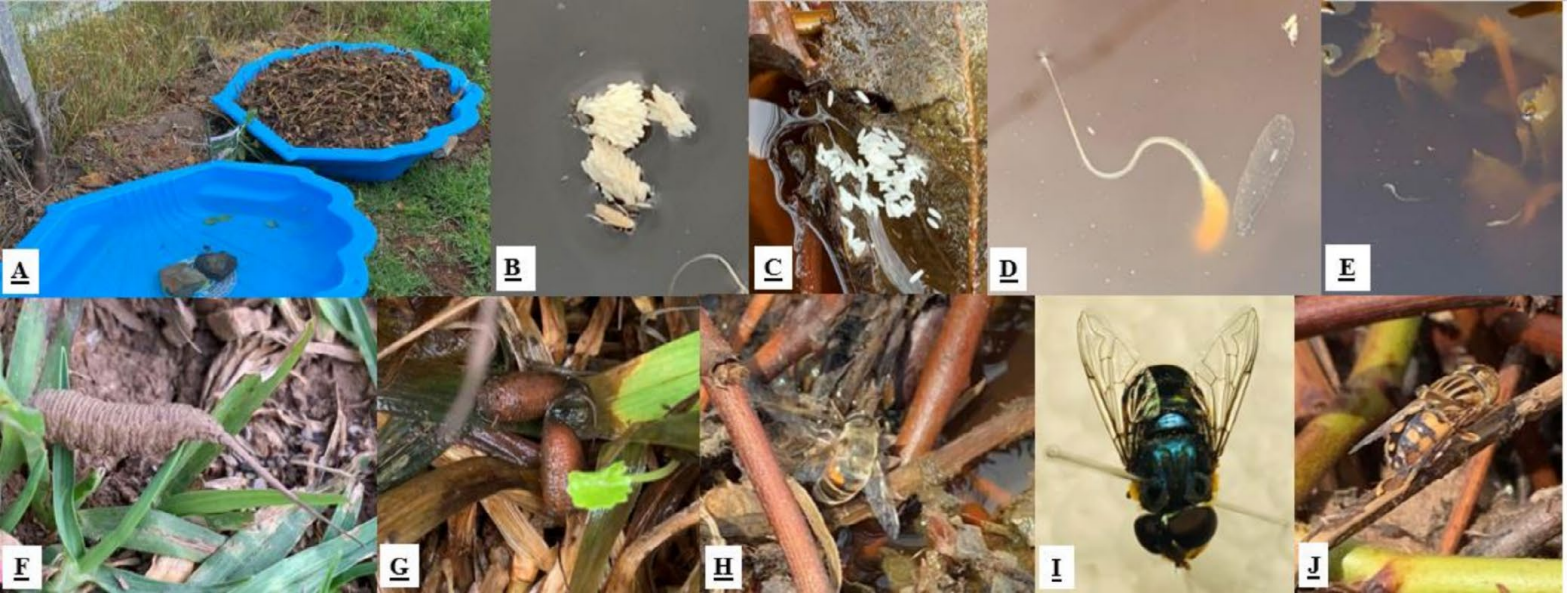
(A) Artificial ponds with two treatments: Water‐only controls and plant‐substrate ponds. (B, C) Eristaline eggs in plant‐substrate ponds. (D, E) Eristaline larvae in the plant‐substrate ponds. (F) Eristaline 3rd instar larva pupating within the adjacent soil and grass. (G) Eristaline pupae in surrounding soil. (H) European drone fly ( *Eristalis tenax* (Linnaeus, 1758) (Diptera: Syrphidae)) laying eggs in the plant‐substrate pond. (I) Green hoverfly (*Austalis copiosa* (Walker, 1852) (Diptera: Syrphidae)) emerged from the collected pupae. (J) Golden native drone fly (*Eristalinus punctulatus* (Macquart, 1847) (Diptera: Syrphidae)) visiting the plant‐substrate pond.

This study was conducted at four locations across one commercial berry farm with blackberry ( *Rubus fruticosus* L.), raspberry ( *Rubus idaeus* L.) and blueberry crops ( *Vaccinium corymbosum* L.), located on the Mid North Coast of New South Wales in Australia (approximately 10 km from Coffs Harbour). Each location was separated by at least 300 m. Two polypropylene ponds (945 mm × 210 mm × 1100 mm) were deployed side by side at each location to evaluate two treatments: water‐only controls (*n* = 4) and plant‐substrate ponds (*n* = 4) containing decomposing blackberry leaves, stems, and water (Figure 1A; see Davis, Schmidt, Harrington, et al. (2023) for further details of design). Plant material was placed in large garbage bags after pruning and stored at the farm for 2 days prior to placement within the artificial ponds to promote decomposition. As we were assessing the diversity of aquatic and semi‐adquatic invertebrate taxa within the artificial ponds, sampling was conducted only in ponds containing water and plant substrate.

All ponds contained equal water volumes (approximately 33 litres each), with water collected from the farm's agricultural hose. The ponds were positioned along farm margins, approximately 10–20 m from the nearest polytunnel, to minimise interference from agricultural machinery. The surrounding berry farms followed standard Australian agricultural practices, including mechanical mowing for weed control, targeted application of herbicides and fungicides and minimal soil disturbance beyond initial bed preparation, consistent with typical berry production systems in Australia (NSW Department of Primary Industries 2020; farm owner, personal communication). All ponds (8 in total) were established simultaneously on 25 September 2024 and remained in the field for up to 43 days, encompassing the austral spring period through to 6 November 2024.

To determine which invertebrate taxa colonised artificial ponds over the sampling period, we documented all visible aquatic and semi‐aquatic invertebrate taxa throughout the trial. Every sampling day (*n* = 8 in total), newly observed taxa were recorded and identified to the family level in the field (~5 species per sampling day). Taxa were classified as either *transient* or *colonising* based on their persistence within the artificial ponds. *Transient taxa* were those observed only 1–3 times during the trial and not present in the final sampling period. *Colonising taxa* were those that remained within the ponds and were still present at the end of the experiment; however, the exact timing of colonisation events may have varied slightly. This interval reflected standard logistical constraints and was chosen to minimise disturbance while ensuring consistent detection of new species. During this sampling period, all berry crops were at different stages of floral bloom; blackberries and raspberries flower from early spring into early/late summer depending on cultivar (Davis et al. 2026), and blueberry flowers from late winter through spring (Hall et al. 2020). This phenological overlap created a prolonged and dynamic foraging environment for adult pollinators throughout the deployment period.

Adult eristaline hoverflies and other flower‐visiting taxa forage across all three berry crops as well as surrounding wild vegetation (Földesi et al. 2016; Bobiwash et al. 2018; Szitár et al. 2022; Aviron et al. 2023; Hanusch et al. 2025). As such, we recorded all invertebrates present in the ponds to capture the full range of taxa with potential access to these resources. Non‐pollinating groups were also included, as they interact with pond habitats through predation, competition, or detrital processing (Wise 1995; Nyffeler and Birkhofer 2017; Whyte and Anderson 2017; Michalko et al. 2019; Lutinski et al. 2024; Ambarningrum et al. 2023; Dawson et al. 2025; de Paula Malheiros et al. 2025; Kheam et al. 2025), and therefore influence overall colonisation patterns. Recording the complete assemblage provided a comprehensive assessment of how the ponds were used within a multi‐crop farm system.

To track larval development in relation to eristaline oviposition timing, all ponds were inspected every 3–4 days after deployment, given that eristaline eggs typically hatch within 72 h after they are laid (Campoy et al. 2020). Plant‐substrate ponds were inspected for 15 min each, with all decaying plant material examined for eggs, larvae and pupae of eristaline flies. Water‐only ponds were inspected for 5 min each. Visual identification of immature stages was feasible because eristaline larvae possess distinctive posterior siphons that protrude from the substrate surface. When present, eggs (Figure 1B,C), larvae (Figure 1D,E), and pupae were counted, with larval instar stages (1st, 2nd and 3rd) counted individually. As eristaline flies pupate after the 3rd larval instar (Figure 1F), systematic searches for pupae were also conducted within the artificial ponds, and within the grass and soil approximately 1 m around the circumference of each pond (Figure 1G,H). Water‐only ponds were excluded from the egg and larval models because no oviposition or larval presence was observed in this treatment at any time point.

Water‐only ponds were removed on day 31 due to the absence of eristaline flies and presence of mosquito larvae, whereas plant‐substrate ponds were retained because they contained 3rd instar eristaline fly larvae, allowing us to continue tracking their development as part of the study's dual focus on both eristaline biology and broader pond colonisation patterns. Plant‐substrate ponds were removed after 43 days once pupae were found. Before pond removal, we collected samples of all invertebrates present within the artificial ponds via individual glass vials, stored the specimens in 70% ethanol, and identified them to the lowest practical taxonomic level using keys or expert aide (Table S1). Eristaline larvae and pupae were collected live and reared to adulthood for species‐level identification at the end of the experiment. To avoid disturbing the pond environment and influencing colonisation or developmental processes, only observations were recorded and no invertebrate sampling was conducted during the experimental period. As a result, it is possible that some taxa present on certain days were not detected. However, no new taxa were recorded or collected on the final day of the trial, suggesting that the majority of colonising groups were consistently observed.

### 2.1 Data Analysis

To analyse taxonomic richness, we quantified the number of invertebrate families detected in each pond on every sampling day. We then fitted generalised linear mixed models (GLMMs) using the ‘lme4’ and ‘glmmTMB’ packages (Brooks et al. 2017) in R version 4.4.2 (R Core Team 2025) to evaluate whether treatment or sampling day influenced richness. Data manipulation and visualisation were performed using the ‘tidyverse’ package (Wickham et al. 2019).

To determine the probability of eristaline eggs and larvae presence depending on the experimental day (41 days in total), we conducted two GLMMs, one for each life cycle stage in R version 4.4.2 (R Core Team 2025) using the ‘glmmTMB’ package. For the response variables, we used the presence or absence (1 and 0, respectively) of ‘eggs’ and ‘larvae’. The fixed effect in both models was ‘days’ (continuous), and ‘location’ (categorical) was used as a random effect. For both GLMMs, we used a binomial distribution. We extracted the predicted probability and confidence intervals (95%) of the eggs and larvae occurring using the ‘effects’ package (Fox and Weisberg 2018, 2019) and plotted the effects using the ‘ggplot2’ package (Wickham 2016). We assessed both models' performance and assumptions using the ‘performance’ package (Lüdecke et al. 2021).

To analyse community‐level patterns, we conducted a PERMANOVA to test whether treatment or sampling day influenced invertebrate community composition. This analysis was performed using the ‘vegan’ package (Oksanen et al. 2025) in R version 4.4.2 (R Core Team 2025). We also attempted to visualise community structure using an nMDS ordination (via ‘vegan’ package), yet the ordination could not be meaningfully interpreted due to the limited number of unique community profiles after removing zero‐only and duplicated rows.

## 3 Results

**FIGURE 2:**
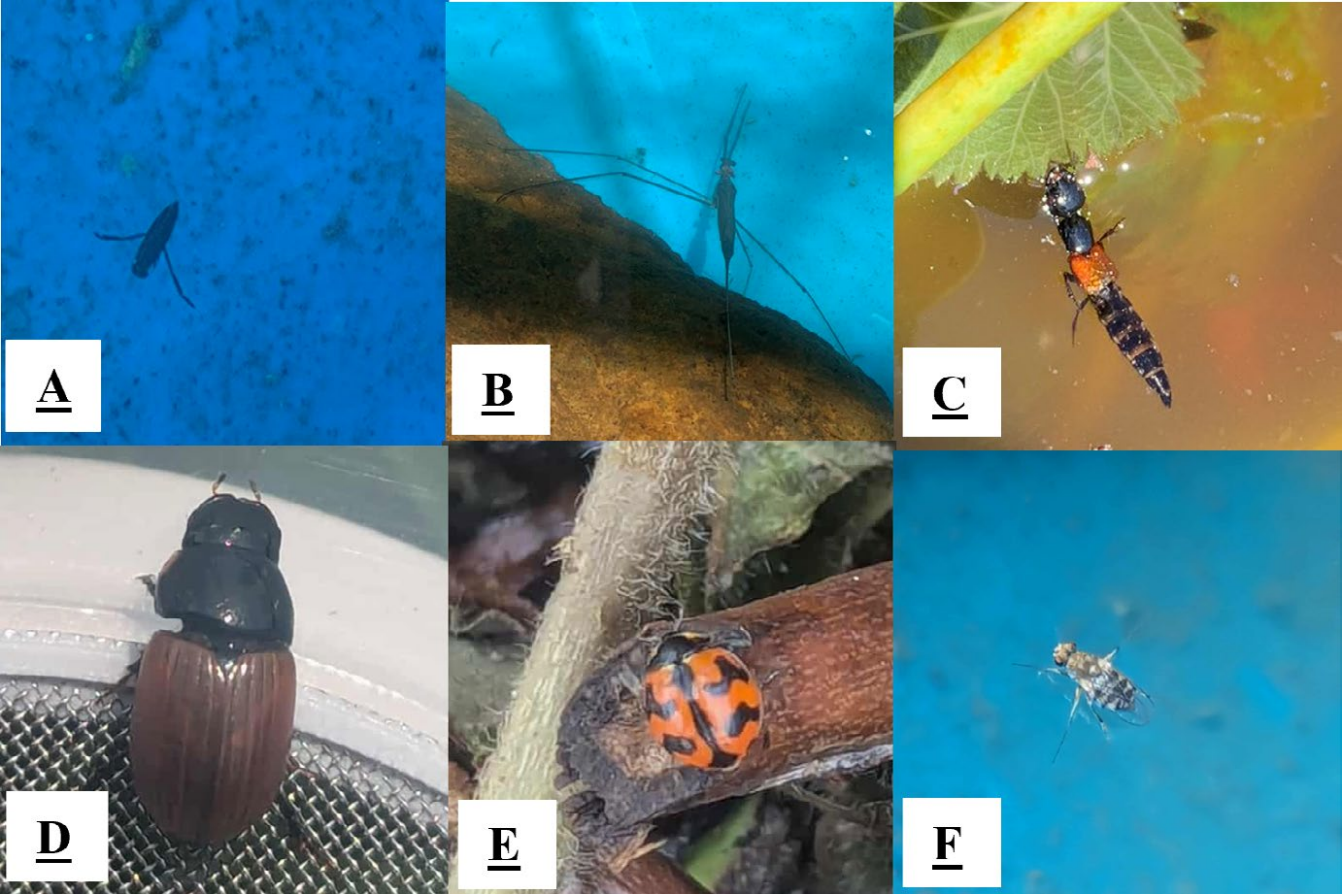
Berry cropping system artificial pond invertebrate taxa: (A) *Rhantus suturalis* (W.S. Macleay, 1825) (Coleoptera: Dytiscidae). (B) *Ranatra dispar* Montandon, 1903 (Hemiptera: Nepidae). (C) *Thyreocephalus rufitarsis* (Fauvel, 1877) (Coleoptera: Staphylinidae). (D) *Aphodius fimetarius* (Linnaeus, 1758) (Coleoptera: Scarabaeidae). (E) *Coccinella transversalis* Fabricius, 1781 (Coleoptera: Coccinellidae). (F) Diptera: Ephydridae Zetterstedt, 1837.

The eight artificial ponds were surveyed for a total of 1280 min, representing the cumulative time spent visually observing invertebrate presence and activity within the ponds. Over this period, 40 invertebrate taxa spanning nine orders were identified. Seven taxa were transient species that appeared briefly but did not persist, while 33 colonised the ponds and remained until the end of the experiment (Table S1). Of the 33 taxa that colonised the artificial ponds, 25 species occurred exclusively within the plant‐substrate pond treatment, eight species were found in the water‐only artificial ponds (Figure 2A,B) and six species were found in both treatments (Table S1). Colonisation began 9 days after deployment, and syrphid flies were the first taxa recorded within plant‐substrate ponds (Table 1).

**TABLE 1:** First recorded occurrence of invertebrate taxa utilising artificial ponds deployed within a commercial berry farm located in the Mid North Coast of New South Wales, Australia. ‘Days’ refers to the number of days after pond deployment when each taxa was first observed. The *‘Plant’* treatment consisted of ponds containing decaying blackberry leaves, stems and water, while the *‘Water’* treatment was the control and contained water only. *‘Order’* is the invertebrate order, *‘Family’* is the taxonomic family and *‘Species’* is the number of species identified within each family. Asterisks (*) indicate taxa that remained within the ponds until the final day of the experiment.

Days	Treatment	Order	Family	Species
9	Plant	Diptera	Syrphidae*	3
13	Plant	Diptera	Sepsidae*	2
13	Plant	Coleoptera	Staphylinidae*	4
13	Both	Diptera	Culicidae *	1
13	Water	Hemiptera	Nepidae	1
13	Water	Coleoptera	Hydrophilidae*	3
18	Plant	Coleoptera	Scarabaeidae*	3
18	Plant	Coleoptera	Coccinellidae	1
20	Plant	Araneae	Lycosidae	1
20	Both	Coleoptera	Dytiscidae*	1
23	Both	Hymenoptera	Apidae	1
23	Plant	Hymenoptera	Formicidae*	1
27	Both	Diptera	Ephydridae*	1
27	Both	Diptera	Empididae*	1
29	Plant	Diptera	Platystomatidae*	1
29	Water	Odonata	Coenagrionidae*	1
29	Water	Odonata	Libellulidae*	1
29	Plant	Diptera	Muscidae	1
31	Both	Blattodea	Rhinotermitidae	1
31	Both	Poduromorpha	Hypogastruridae*	1
31	Plant	Araneae	Araneidae*	1
31	Plant	Araneae	Tetragnathidae*	1
31	Plant	Araneae	Theridiidae*	1
31	Plant	Coleoptera	Mycetophagidae*	1
31	Plant	Coleoptera	Ptiliidae*	1
31	Plant	Hemiptera	Cydnidae*	1
31	Water	Coleoptera	Chrysomelidae*	1
31	Water	Hemiptera	Cicadellidae*	1
31	Water	Stylommatophora	Limacidae*	1
34	Plant	Diptera	Calliphoridae	1

Taxa represented aquatic, semi‐aquatic, and terrestrial groups, including Araneae (spiders), Coleoptera (beetles; Figure 2C–E), Collembola (springtails), Diptera (flies; Figure 2F), Hemiptera (true bugs), Hymenoptera (ants and bees), Odonata (dragonflies and damselflies) and Stylommatophora (slugs).

Of the seven transient taxa recorded, four were observed only in the plant‐substrate treatment, one occurred exclusively in the water‐only treatment and two utilised both treatments. The water scorpion *Ranatra dispar* (Hemiptera: Nepidae) was recorded in a water‐only pond 13 days after deployment but was not detected during subsequent sampling periods (Figure 2B). Also, the small transverse lady beetle (*Coccinella transversalis* Fabricius, 1781) was recorded within the plant‐substrate pond on day 18 after deployment (Figure 2E). Similarly, a wolf spider (Araneae: Lycosidae) was observed on day 20 in one plant‐substrate pond but did not reappear. Honey bees (*Apis mellifera (Linnaeus, 1758)*) were observed drinking water from both pond types on days 23, 27 and 29 post pond deployment. In addition, bush flies (Diptera: Muscidae) were seen landing on the pond edges and substrate within the ponds 29 days after deployment.

**FIGURE 3:**
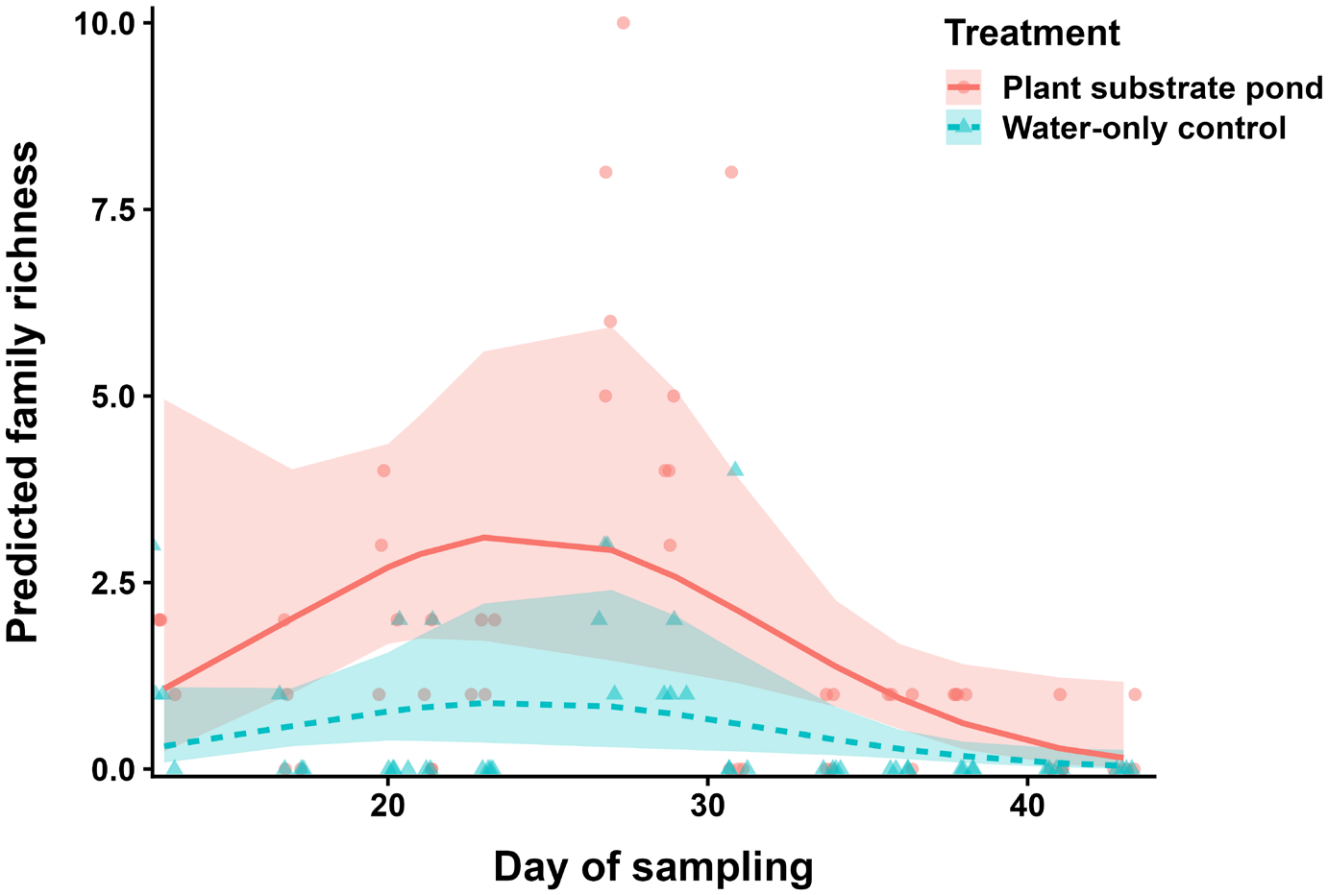
Predicted mean family richness across the 45‑day sampling period for plant‑substrate ponds (solid red line) and water‑only controls (dashed blue line). Shaded ribbons represent 95% confidence intervals, and points show raw observations.

Plant‐substrate ponds supported significantly higher family richness than water‐only controls (*p* < 0.0001; Figure 3). Across the sampling period, predicted richness was consistently greater in the plant‐substrate treatment, with model‐predicted values and observed data points showing clear separation between treatments (Table S2). Richness in both treatments rose early in the sampling period, peaked around mid‐season and declined towards the end, but the magnitude of richness remained markedly higher in plant‐substrate ponds at all stages.

We found 30 families visiting the ponds. Insect community composition varied significantly through time (PERMANOVA, Bray‐Curtis, *p* = 0.001; Table S3), while differences between plant‐substrate ponds and water‐only controls were not statistically significant (*p* = 0.31; Table S3). Insect communities inhabiting ponds showed substantial overlap in community composition between treatments, with many sampling occasions characterised by identical family‐level assemblages.

While such incidental visitors were sporadic, eristaline flies were among the earliest and most consistent colonisers of the artificial plant‐substrate ponds. Three eristaline species were recorded: the European drone fly ( *Eristalis tenax* ; Figure 1H), the green hoverfly (*Austalis copiosa* (Walker, 1852); Figure 1I) and the golden Australian native drone fly (*Eristalinus punctulatus*; Figure 1J). Eristaline eggs, larvae or pupae were observed exclusively in plant‐substrate ponds and never in water‐only ponds.

**FIGURE 4:**
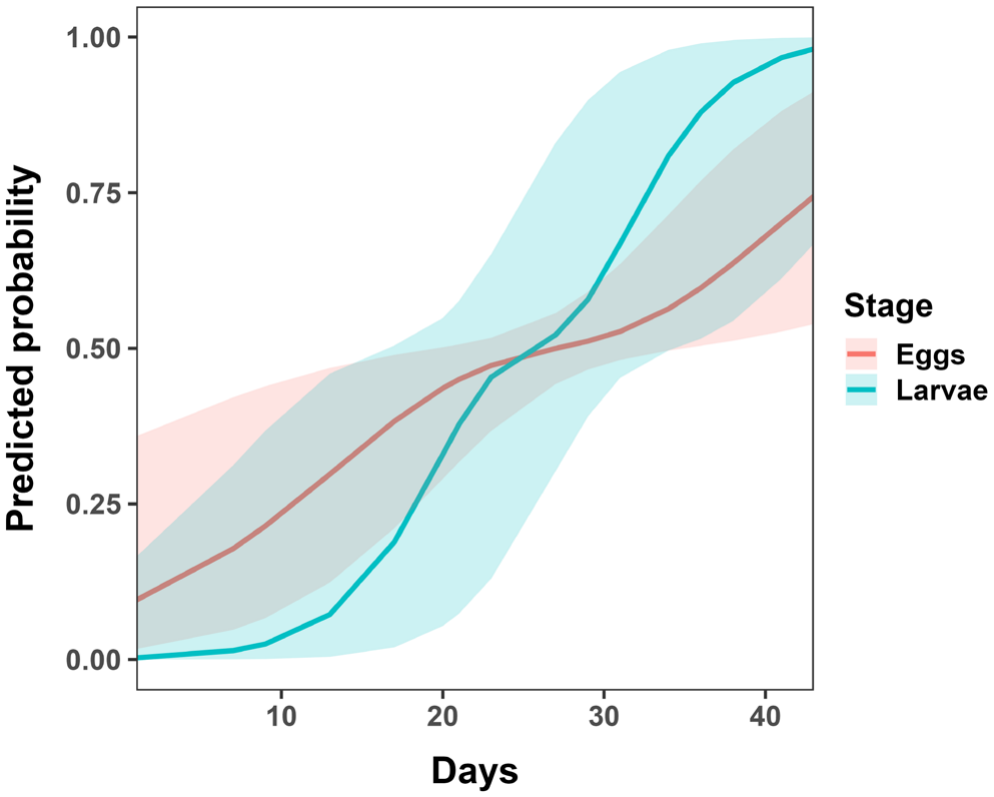
The predicted probability of eristaline fly eggs and larvae occurring in plant‐substrate ponds according to days after pond deployment. The x‐axis represents days after deployment of artificial ponds, and the y‐axis represents the predicted probability of eggs and larvae occurring in plant‐substrate ponds. Lines represent the mean model probability and shaded error regions represent 95% CI derived from the GLMMs.

Eggs were first observed 9 days after deployment, followed by larvae on day 17, and pupae on day 38 in adjacent soil and grass near the ponds at location four (Table S4). Model predictions indicated that egg occurrence in plant‐substrate ponds increased steadily over time, reaching a 75% likelihood by day 40, while larval occurrence approached nearly 100% likelihood (Figure 4).

## 4 Discussion

This study demonstrates that artificial pond habitats can attract and support a taxonomically and functionally diverse assemblage of invertebrates within a commercial berry farm. Within 25 days of deployment, the ponds were utilised by a range of functionally important taxa, including known predators (e.g., lady beetles), pollinators (e.g., eristaline flies, honey bees) and nutrient recyclers (e.g., eristaline flies, springtails). The plant‐substrate ponds supported substantially higher diversity, hosting three times more colonising taxa than the water‐only controls. Notably, eristaline hoverflies were among the earliest and most persistent colonisers, appearing within 9 days of pond deployment, demonstrating that this habitat type can contribute to the provision of pollination and nutrient‐cycling ecosystem services within the system. Although some transient visitors (e.g., lady beetles, honey bees, wolf spiders) were detected briefly, their presence suggests that even small artificial water bodies can provide critical habitat elements such as supplementary refuge or alternative foraging points within intensively managed farms (Iuliano et al. 2024). However, whether this enhanced invertebrate diversity translates into greater occurrence or ecosystem services within the system, provision in crop fields remains unknown and will require future field‐scale trials. Collectively, these findings reveal that even simple, low‐disturbance pond habitats can meaningfully enhance invertebrate diversity and functional contributions in horticultural landscapes.

Several species recorded within the ponds may have beneficial or detrimental effects in agricultural landscapes, depending on their ecological roles and interactions. A number of colonising and transient species may enhance on‐farm ecological functioning through pollination, nutrient cycling, or pest regulation (Whyte and Anderson 2017; Lutinski et al. 2024; Ambarningrum et al. 2023; Dawson et al. 2025). For example, sepsid flies (Diptera: Sepsidae), common floral visitors of carrot umbels (Dawson et al. 2025), and the ant *Tetramorium bicarinatum* (*Nylander, 1846*) (*Hymenoptera: Formicidae*), a known biological control agent (Lutinski et al. 2024), both colonised the ponds. Predatory transient taxa such as spiders and lady beetles were also detected; while these taxa can suppress pests (de Paula Malheiros et al. 2025; Kheam et al. 2025), spiders may also predate upon beneficial insects (Wise 1995; Nyffeler and Birkhofer 2017; Whyte and Anderson 2017; Michalko et al. 2019). Detritivores such as springtails (Collembola Lubbock, 1871), which promote nutrient cycling and soil health (Ambarningrum et al. 2023), also colonised the ponds. At the same time, several pest species, including mosquitoes (Diptera: Culicidae), *Chaetocnema* beetles (Coleoptera: Chrysomelidae), the African black beetle *Heteronychus arator (Fabricius, 1775)*, leafhoppers (Hemiptera: Cicadellidae) and slugs (Limacidae), were also recorded, all of which can damage crops or transmit pathogens (Mathys and Smith 1984; Weintraub et al. 2019; Lv et al. 2024; Heddle et al. 2025). These results highlight the trade‐offs between fostering beneficial invertebrates and inadvertently creating refuges for pests or predators of beneficials, which may impact growers' adoption of ponds as a non‐floral resource to enhance biodiversity benefits.

Flower visitors observed developing within the artificial ponds included hoverflies *E. tenax* and *E. punctulatus* , recognised as effective pollinators of various commercial crops (Howlett and Gee 2019; Davis, Schmidt, Harrington, et al. 2023; Davis, Schmidt, Santos, et al. 2023). Eristaline flies oviposited and their larvae emerged within the artificial ponds, while pupation occurred in the surrounding soil. This reliance on multiple microhabitats aligns with broader evidence that artificial ponds can enhance connectivity in fragmented agricultural landscapes by acting as stepping‐stone habitats for species movement (Cannucci et al. 2025; Briggs et al. 2019). Consistent with wider ecological patterns, local pond attributes (often shaped by site‐specific management) can exert a stronger influence on biodiversity than landscape‐scale factors (Cannucci et al. 2025), highlighting the importance of considering pond‐level conditions when interpreting biological responses. Together, these findings suggest that variations of integrated aquatic‐terrestrial pond designs could support the full life cycle of some fly pollinators within managed horticultural systems.

Some pond taxa, including eristaline flies, suffered from early‐stage mortality, potentially due to predation by ants, rove beetles or diving beetles. However, the detection of pupae within 38 days indicates successful development can occur under field conditions. Since flies, particularly hoverflies (Diptera: Syrphidae), are increasingly valued for their role in commercial crop pollination due to their widespread occurrence and persistent use of floral resources (Dag and Gazit 2000; Fajardo et al. 2008; Ssymank et al. 2008; Reddy and Sreedevi 2016; Alqarni et al. 2017; Doyle et al. 2020, 2025; Sánchez et al. 2022; Davis, Bickel, Saunders, and Rader 2023; Davis, Schmidt, Harrington, et al. 2023; Davis et al. 2025; Dawson et al. 2025), they represent a promising target group for habitat enhancement in agricultural systems. Moreover, artificial ponds offer habitat for native and poorly studied species, including *Austalis copiosa*, potentially supporting their full life cycles and contributing to local biodiversity.

Overall, artificial ponds offer a simple, potentially scalable approach to enhancing on‐farm biodiversity and supporting pollinating flies. While many taxa were likely beneficial, others may act as pests (such as mosquitos), underscoring the need to monitor pond colonisation and balance ecological benefits and risks when deploying such habitats in commercial settings. Enhancing farm biodiversity by supporting a high diversity of pond invertebrates and efficient fly pollinators may result in agronomic and ecological benefits. Efficient pollinator species could contribute to improved crop yields and higher market quality. Additionally, artificial ponds attract predatory beneficial invertebrates that provide natural pest control, reducing the need for chemical inputs. These systems also facilitate more sustainable management of organic waste, promoting nutrient recycling and overall farm resilience. Future research could focus on increasing understanding of the trophic roles of each of the taxa using the ponds, identification of pest and pathogen concerns and a greater understanding of the function of these habitats in enhancing ecosystem service delivery, particularly crop pollination.

## Author Contributions

**Jelena Preradovic:** conceptualisation (lead); data curation (lead); formal analysis (lead); investigation (lead); methodology (equal); visualisation (lead); writing – original draft (lead); writing – review and editing (equal). **Romina Rader:** conceptualisation (equal); funding acquisition (lead); methodology (equal); project administration (equal); resources (equal); supervision (equal); validation (equal); writing – review and editing (equal). **Blake M. Dawson:** conceptualisation (equal); formal analysis (equal); supervision (equal); visualisation (equal); writing – review and editing (equal). **Lena A. Schmidt:** conceptualisation (supporting); methodology (supporting); supervision (equal); validation (equal); writing – review and editing (equal). **Raylea Rowbottom:** writing – review and editing (equal). **Abby E. Davis:** conceptualisation (equal); data curation (equal); investigation (equal); methodology (equal); supervision (equal); validation (equal); writing – review and editing (equal).

## Funding

This work was funded by the Future Food Systems Top‐up PhD Scholarship (number: P2‐033). The research was partly funded by Hort Innovation (MT22007), using the raspberry, blackberry, blueberry research and development levies, contributions from the Australian Government and an Australian Research Council Future Fellowship (FT210100851) awarded to Romina Rader. Open access publishing was facilitated by the University of New England, as part of the Wiley–University of New England agreement via the Council of Australian University Librarians.

## Conflicts of Interest

Raylea Rowbottom was employed by seedPurity Pty/Ltd., while all other remaining authors declare no conflicts of interest.

## Supporting information


**Table S1:** Invertebrate species identified from artificial pond habitats (plant‐substrate and water‐only treatments) deployed in a commercial berry farm near Coffs Harbour, New South Wales, Australia. The plant‐substrate pond contained blackberry leaves and stems collected after pruning (plants were collected in bags 2 days before being deployed in the pools) and approximately 33 L of water, while the water‐only control treatment was filled with equal amounts of water. Single asterisks (*) indicate *colonising taxa* which remained within the ponds until the final day of the experiment; triple asterisks (***) indicate *transient taxa* which were observed only once or a few times during the 43‐day trial and not present in the final sampling period. Species presence (1) and absence (0) are shown for each of the eight deployed ponds (four per treatment). Numbers 1–4 under ‘Plant‐substrate pond’ and ‘Water‐only control’ denoting the individual replicate ponds. Lowercase letters denote species categories: (b) beneficial; (p) pest; and (no info.) no information available. The ‘Resource for identification’ column indicates the resource used to identify the insect specimen to the lowest taxonomic level possible.
**Table S2:** Fixed‐effects estimates from the conditional model examining treatment and sampling‐day effects on family richness.
**Table S3:** Results of the PERMANOVA showing the effects of treatment and sampling day on multivariate community structure.
**Table S4:** First appearance of Eristaline (Diptera: Syrphidae) fly eggs, larvae and pupae in artificial pond habitats related to days and location. Each location (1; 2; 3; 4) had two ponds (Plant‐substrate pond and Water‐only control) positioned 10–20 m from the nearest polytunnels. Locations were established at least 300 m between them. Days‐ indicates the 43‐day period during which ponds remained in the fields, and the specific day post‐deployment when eggs, larvae, and pupae were first recorded.

## Data Availability

Data available via OSF: https://osf.io/jz4sh/overview?view_only=05f746ef84034fc1a47e17d08736b2c5.
