# Supplementary material for: Artificial Pond Habitats Placed in an Australian Berry Farm Support Invertebrate Diversity Including Pollinating Flies
Source: Ecol Evol. 2026 Apr 7;16(4):e73423. doi: 10.1002/ece3.73423 (PMC13058237; doi:10.1002/ece3.73423)
Supplement: Supplementary file 1 — Table S1: Invertebrate species identified from artificial pond habitats (plant‐substrate and water‐only treatments) deployed in a commercial berry farm near Coffs Harbour, New South Wales, Australia. The plant‐substrate pond contained blackberry leaves and stems collected after pruning (plants were collected in bags 2 days before being deployed in the pools) and approximately 33 L of water, while the water‐only control treatment was filled with equal amounts of water. Single asterisks (*) indicate colonising taxa which remained within the ponds until the final day of the experiment; triple asterisks (***) indicate transient taxa which were observed only once or a few times during the 43‐day trial and not present in the final sampling period. Species presence (1) and absence (0) are shown for each of the eight deployed ponds (four per treatment). Numbers 1–4 under ‘Plant‐substrate pond’ and ‘Water‐only control’ denoting the individual replicate ponds. Lowercase letters denote species categories: (b) beneficial; (p) pest; and (no info.) no information available. The ‘Resource for identification’ column indicates the resource used to identify the insect specimen to the lowest taxonomic level possible. Table S2: Fixed‐effects estimates from the conditional model examining treatment and sampling‐day effects on family richness. Table S3: Results of the PERMANOVA showing the effects of treatment and sampling day on multivariate community structure. Table S4: First appearance of Eristaline (Diptera: Syrphidae) fly eggs, larvae and pupae in artificial pond habitats related to days and location. Each location (1; 2; 3; 4) had two ponds (Plant‐substrate pond and Water‐only control) positioned 10–20 m from the nearest polytunnels. Locations were established at least 300 m between them. Days‐ indicates the 43‐day period during which ponds remained in the fields, and the specific day post‐deployment when eggs, larvae, and pupae were first recorded. [file ECE3-16-e73423-s001.docx]

**Supplemental material**

**Artificial pond habitats placed in an Australian berry farm support invertebrate diversity including pollinating flies**

Supplementary Table 1. Invertebrate species identified from artificial pond habitats (plant-substrate and water-only treatments) deployed in a commercial berry farm near Coffs Harbour, New South Wales, Australia. The plant-substrate pond contained blackberry leaves and stems collected after pruning (plants were collected in bags two days before being deployed in the pools) and approximately 33 liters of water, while the water-only control treatment was filled with equal amounts of water. Single asterisks (*) indicate colonizing taxa which remained within the ponds until the final day of the experiment; triple asterisks (***) indicate transient taxa -which were observed only once or a few times during the 43-day trial and not present in the final sampling period. Species presence (1) and absence (0) are shown for each of the eight deployed ponds (four per treatment). Numbers 1-4 under “Plant-substrate pond” and “Water-only control” denoting the individual replicate ponds. Lowercase letters denote species categories: (b) beneficial; (p) pest; and (no info.) no information available. The “Resource for identification” column indicates the resource used to identify the insect specimen to the lowest taxonomic level possible.

| **Order** | **Family** | **Species** | **Plant-substrate pond** | | | | **Water-only control** | | | | **Resources for identification** |
| --- | --- | --- | --- | --- | --- | --- | --- | --- | --- | --- | --- |
|  |  |  | No. 1 | No. 2 | No. 3 | No. 4 | No. 1 | No. 2 | No. 3 | No. 4 |  |
| Araneae | Araneidae | *Socca pustulosa* (Walckenaer, 1842) * (b) | 1 | 0 | 0 | 0 | 0 | 0 | 0 | 0 | https://keys.lucidcentral.org/search/key-to-spider-subfamilies-of-australia/ |
| Araneae | Lycosidae | sp 1 ***** (b) | 1 | 0 | 0 | 0 | 0 | 0 | 0 | 0 | https://keys.lucidcentral.org/search/key-to-spider-subfamilies-of-australia/ |
| Araneae | Tetragnathidae | *Tetragnatha* Latreille, 1804 sp 1 * (b) | 0 | 0 | 1 | 0 | 0 | 0 | 0 | 0 | https://keys.lucidcentral.org/search/key-to-spider-subfamilies-of-australia/ |
| Araneae | Theridiidae Sundevall, 1833 | sp 1 * (b) | 0 | 0 | 1 | 0 | 0 | 0 | 0 | 0 | https://keys.lucidcentral.org/search/key-to-spider-subfamilies-of-australia/ |
| Blattodea | Rhinotermitidae Froggatt, 1897 | sp 1 *** (no info.) | 1 | 1 | 1 | 1 | 1 | 1 | 1 | 1 | https://bie.ala.org.au/species/ALA_DR22913_2447#gallery |
| Coleoptera | Chrysomelidae | *Chaetocnema* Stephens, 1831 sp 1 * (p) | 0 | 0 | 0 | 0 | 0 | 0 | 1 | 0 | https://www.inaturalist.org/observations/268328321 |
| Coleoptera | Coccinellidae | *Coccinella transversalis* Fabricius, 1781 *** (b) | 1 | 0 | 0 | 0 | 0 | 0 | 0 | 0 | Expert identification |
| Coleoptera | Dytiscidae | *Rhantus suturalis* (W.S. Macleay, 1825) * (no info.) | 0 | 0 | 0 | 1 | 1 | 0 | 0 | 0 | https://www.inaturalist.org/observations/246055092 |
| Coleoptera | Hydrophilidae | *Helochares* Mulsant, 1844 sp 1 * (b) | 0 | 0 | 1 | 0 | 0 | 0 | 0 | 0 | https://www.inaturalist.org/taxa/492694-Helochares |
| Coleoptera | Hydrophilidae | *Megasternini* Mulsant, 1844 sp 1 * (no info.) | 1 | 1 | 1 | 0 | 0 | 0 | 0 | 0 | https://www.inaturalist.org/observations/268325548 |
| Coleoptera | Hydrophilidae | *Megasternini* sp 2 * (b) | 0 | 1 | 0 | 0 | 0 | 0 | 0 | 0 | https://www.inaturalist.org/observations/268325548 |
| Coleoptera | Mycetophagidae Leach, 1815 | sp 1 * (no info.) | 0 | 0 | 1 | 0 | 0 | 0 | 0 | 0 | https://keys.lucidcentral.org/search/beetles-of-the-world/ |
| Coleoptera | Ptiliidae Motschulsky / Erichson, 1845 | sp 1 * (no info.) | 1 | 1 | 0 | 1 | 0 | 0 | 0 | 0 | https://keys.lucidcentral.org/search/beetles-of-the-world/ |
| Coleoptera | Scarabaeidae | *Onitis alexis* Klug, 1835 * (b) | 0 | 0 | 0 | 1 | 0 | 0 | 0 | 0 | https://www.kclg.org.au/images/dungbeetles/DUNG%20BEETLE%20IDENTIFICATION%20GUIDE.pdf |
| Coleoptera | Scarabaeidae | *Aphodius fimetarius* (Linnaeus, 1758) * (b) | 1 | 0 | 0 | 0 | 0 | 0 | 0 | 0 | https://agriculture.vic.gov.au/support-and-resources/funds-grants-programs/on-farm-demonstrations-program/aphodius-fimetarius |
| Coleoptera | Scarabaeidae | *Heteronychus* *arator* (Fabricius, 1775) * (p) | 1 | 0 | 0 | 0 | 0 | 0 | 0 | 0 | https://www.inaturalist.org/observations/269045314 |
| Coleoptera | Staphylinidae | *Thyreocephalus rufitarsis* (Fauvel, 1877) * (b) | 1 | 1 | 0 | 1 | 0 | 0 | 0 | 0 | https://www.inaturalist.org/observations/269033849 |
| Coleoptera | Staphylinidae | *Oxytelini sp.* 1 * (b) | 0 | 1 | 0 | 0 | 0 | 0 | 0 | 0 | https://www.inaturalist.org/observations/269067503 |
| Coleoptera | Staphylinidae | sp 3 * (no info.) | 1 | 1 | 0 | 1 | 0 | 0 | 0 | 0 | https://keys.lucidcentral.org/search/beetles-of-the-world/ |
| Coleoptera | Staphylinidae | sp 4 * (no info.) | 1 | 0 | 0 | 0 | 0 | 0 | 0 | 0 | https://keys.lucidcentral.org/search/beetles-of-the-world/ |
| Collembola | Hypogastruridae | *Hypogastrura* Bourlet, 1839 sp 1 *** (b) | 1 | 1 | 1 | 0 | 1 | 1 | 1 | 0 | https://keys.lucidcentral.org/search/key-to-order-superfamily-of-australian-collembola/ |
| Diptera | Calliphoridae | *Lucilia* spp. *** (b) | 1 | 0 | 0 | 0 | 0 | 0 | 0 | 0 | Expert identification |
| Diptera | Culicidae Meigen, 1818 | sp 1 (larvae) * (no info.) | 1 | 1 | 1 | 1 | 1 | 1 | 0 | 1 | https://www.mdfrc.org.au/bugguide/display.asp?type=5&class=17&SubClass=&Order=7&family=251&genus=&species=&couplet=0&fromcouplet=1 |
| Diptera | Culicidae | sp 1 (pupae) * (no info.) | 0 | 1 | 1 | 0 | 1 | 1 | 0 | 1 | https://www.mdfrc.org.au/bugguide/display.asp?type=5&class=17&SubClass=&Order=7&family=251&genus=&species=&couplet=0&fromcouplet=1 |
| Diptera | Empididae Latreille, 1804 | sp 1 (larvae) * (b) | 0 | 1 | 1 | 0 | 0 | 1 | 0 | 0 | Marshall, S. A. (2012). Flies: The Natural History and Diversity of Diptera. Firefly Books. ISBN 9781770851009. |
| Diptera | Ephydridae Zetterstedt, 1837 | sp 1 * (no info.) | 1 | 0 | 0 | 1 | 0 | 0 | 1 | 1 | Marshall, S. A. (2012). Flies: The Natural History and Diversity of Diptera. Firefly Books. ISBN 9781770851009. |
| Diptera | Muscidae  Latreille, 1802 | sp 1 *** (b) | 1 | 0 | 0 | 0 | 0 | 0 | 0 | 0 | Marshall, S. A. (2012). Flies: The Natural History and Diversity of Diptera. Firefly Books. ISBN 9781770851009. |
| Diptera | Platystomatidae Schiner, 1862 | sp 1 * (no info.) | 1 | 0 | 0 | 0 | 0 | 0 | 0 | 0 | Marshall, S. A. (2012). Flies: The Natural History and Diversity of Diptera. Firefly Books. ISBN 9781770851009. |
| Diptera | Sepsidae | *Parapalaeosepsis plebeia* (De Meijere, 1906) * (b) | 1 | 1 | 1 | 1 | 0 | 0 | 0 | 0 | https://keys.lucidcentral.org/keys/v3/Carrion_flies_of_Australia/Parapalaeosepsis_plebeia.htm |
| Diptera | Sepsidae | *Lasionemopoda hirsuta* (De Meijere, 1906) * (b) | 0 | 0 | 1 | 1 | 0 | 0 | 0 | 0 | https://sepsidnet.biodiversity.online/species/Sepsidae-000233-hirsuta |
| Diptera | Syrphidae | *Austalis copiosa*(Walker, 1852) * (b) | 1 | 1 | 1 | 1 | 0 | 0 | 0 | 0 | Expert identification |
| Diptera | Syrphidae | *Eristalis tenax* (Linnaeus, 1758) * (b) | 1 | 1 | 1 | 1 | 0 | 0 | 0 | 0 | Expert identification |
| Diptera | Syrphidae | *Eristalinus punctulatus (Macquart, 1847)* * (b) | 1 | 1 | 1 | 1 | 0 | 0 | 0 | 0 | Expert identification |
| Hemiptera | Cicadellidae | *Bakeriana nigra* Evans, 1966 * (p) | 0 | 0 | 0 | 0 | 1 | 0 | 0 | 0 | https://idtools.dpi.nsw.gov.au/keys/leafhop/eurymelinae/bakerian.htm |
| Hemiptera | Cydnidae | *Geotomini* Wagner, 1963 sp 1 * (no info.) | 1 | 0 | 0 | 0 | 0 | 0 | 0 | 0 | https://www.inaturalist.org/observations/269039362 |
| Hemiptera | Nepidae | *Ranatra dispar* Montandon, 1903*** (b) | 0 | 0 | 0 | 0 | 1 | 0 | 0 | 0 | https://www.inaturalist.org/observations/246055092 |
| Hymenoptera | Apidae | *Apis mellifera* Linnaeus, 1758***** (b) | 0 | 0 | 0 | 1 | 0 | 1 | 0 | 0 | Expert identification |
| Hymenoptera | Formicidae | *Tetramorium* *bicarinatum* (Nylander, 1846) * (b) | 1 | 0 | 0 | 1 | 0 | 0 | 0 | 0 | https://www.antwiki.org/wiki/Tetramorium_bicarinatum |
| Odonata | Coenagrionidae | sp 1 (adult and nymphs) * (b) | 0 | 0 | 0 | 0 | 1 | 0 | 0 | 0 | Theischinger, G., Hawking, J., & Orr, A. (2021). The Complete Field Guide to Dragonflies of Australia (2nd ed.). CSIRO Publishing. ISBN 9781486313747. |
| Odonata | Libellulidae | *Rhyothemis* *graphiptera* (Rambur, 1842) * (b) | 0 | 0 | 0 | 0 | 1 | 0 | 0 | 0 | Theischinger, G., Hawking, J., & Orr, A. (2021). The Complete Field Guide to Dragonflies of Australia (2nd ed.). CSIRO Publishing. ISBN 9781486313747. |
| Stylommatophora | Limacidae | sp 1 * (p) | 0 | 0 | 1 | 1 | 0 | 0 | 0 | 0 | https://keys.lucidcentral.org/search/terrestrial-mollusc-tool/ |

Supplementary Table 2. Fixed‑effects estimates from the conditional model examining treatment and sampling‑day effects on family richness. Significance codes: 0 ‘’ 0.001 ‘’ 0.01 ‘’ 0.05 ‘.’ 0.1 ‘ ’ 1.

| Conditional model: | | | | |
| --- | --- | --- | --- | --- |
|  | Estimate | Std. Error | z value | Pr(>\|z\|) |
| (Intercept) | -3.866003 | 3.443394 | -1.123 | 0.26155 |
| treatment water-only control | -1.252755 | 0.384284 | -3.260 | 0.00111 ** |
| poly (day of sampling, 2, raw = TRUE)1 | 0.414421 | 0.273177 | 1.517 | 0.12926 |
| poly (day of sampling, 2, raw = TRUE)2 | -0.008569 | 0.005029 | -1.704 | 0.08837 |

Supplementary Table 3. Results of the PERMANOVA showing the effects of treatment and sampling day on multivariate community structure. Significance codes: 0 ‘***’ 0.001 ‘**’ 0.01 ‘*’ 0.05 ‘.’ 0.1 ‘ ’ 1

|  | Df | Sum Of Sqs | R2 | F | Pr(>F) |
| --- | --- | --- | --- | --- | --- |
| treatment | 1 | 1.5583 | 0.07534 | 4.4272 | 0.307 |
| day of sampling | 1 | 2.0485 | 0.09904 | 5.8200 | 0.001 *** |
| Residual | 48 | 16.8953 | 0.81685 |  |  |
| Total | 50 | 20.6835 | 1.00000 |  |  |

Supplementary Table 4. First appearance of Eristaline (Diptera: Syrphidae) fly eggs, larvae and pupae in artificial pond habitats related to days and location. Each location (1; 2; 3; 4) had two ponds (Plant-substrate pond and Water-only control) positioned 10 to 20 metres from the nearest polytunnels. Locations were established at least 300 m between them. Days- indicates the 43-day period during which ponds remained in the fields, and the specific day post-deployment when eggs, larvae, and pupae were first recorded.

| **Location** | **Days to first observation** | | |
| --- | --- | --- | --- |
|  | **Eggs** | **Larvae** | **Pupae** |
| **1** | 27 | 31 | 0 |
| **2** | 20 | 23 | 0 |
| **3** | 29 | 31 | 0 |
| **4** | 9 | 17 | 38 |
